# Supplementary material for: Analysis of PPARγ Signaling Activity in Psoriasis
Source: Int J Mol Sci. 2021 Aug 10;22(16):8603. doi: 10.3390/ijms22168603 (PMC8395241; doi:10.3390/ijms22168603)
Supplement: Supplementary file 1 [file ijms-22-08603-s001.zip › Supplemental materials_Analysis of PPARg signaling activity in psoriasis/Pathway models/Models images and html files/PPARG inhibits phenotypes assotiated with psoriasis/238645.html]

interstitial fibrosis


# Disease interstitial fibrosis

|  |  |
| --- | --- |
| URN | urn:agi-disease:kidney%20fibrosis |
| Total Entities | 1 |
| Connectivity | 3313 |
| Name | interstitial fibrosis |

---

|  |  |
| --- | --- |
| ChildConcepts | experimental renal fibrosis |

---

|  |  |
| --- | --- |
| Pathway | Cyclosporine Induced Nephrotoxicity |
|  | cypa |
|  | PPARG inhibits phenotypes assotiated with psoriasis |
|  | Neighbors of interstitial fibrosis |

---

|  |  |
| --- | --- |
| MedScan ID | 9001900 |

---

|  |  |
| --- | --- |
| Alias | renal fibroses |
|  | fibrotic kidneys |
|  | kidney fibrosis |
|  | tubulointerstitial fibrosis |
|  | fibrotic renal |
|  | fibrotic kidney |
|  | fibrosis in kidney |
|  | renal interstitial fibrosis |
|  | interstitial fibrosis |
|  | glomerular fibrosis |
|  | renal fibrosis |
|  | glomerulus fibrosis |
|  | interstitial renal fibrosis |
|  | kidney interstitial fibrosis |
|  | fibrotic glomerulus |

---
